# Supplementary material for: A novel ultrasound-guided mouse model of sudden cardiac arrest
Source: PLoS One. 2020 Dec 4;15(12):e0237292. doi: 10.1371/journal.pone.0237292 (PMC7717537; doi:10.1371/journal.pone.0237292)
Supplement: S1 Table — (DOCX) [file pone.0237292.s001.docx]

**S1 Table. Physiologic and Surgical Characteristics between Sexes.**

| **Sham Mice** | | | |
| --- | --- | --- | --- |
|  | **Female (±SEM)** | **Male (±SEM)** | **p-value** |
| Age (d) | 58.7±1.2 | 55.8±0.4 | 0.04 |
| Weight (g) | 19.4±0.5 | 25.5±1.2 | 0.004 |
| Baseline EF (%) | 58.5±2.0 | 60.8±2.2 | 0.44 |
| 1 d EF (%) | 59.6±3.0 | 60.1±2.5 | 0.89 |
| Initial Body Temp (°C) | 35.7±0.2 | 35.8±0.3 | 0.69 |
| Surgical Survival | 10/11 | 9/9 | n/a |
| **Arrest Mice** | | | |
|  | **Female (±SEM)** | **Male (±SEM)** | **p-value** |
| Age (d) | 57.6±0.8 | 57.9±0.7 | 0.72 |
| Weight (g) | 20.4±0.7 | 25.2±0.3 | <0.001 |
| Baseline EF (%) | 60.0±1.4 | 59.7±1.1 | 0.87 |
| 1 d EF (%) | 37.8±4.9 | 41.8±4.5 | 0.56 |
| Initial Body Temp (°C) | 35.7±0.2 | 35.4±0.3 | 0.42 |
| ROSC Body Temp (°C) | 35.6±0.3 | 35.5±0.3 | 0.86 |
| CPR Duration (min) | 1.3±0.2 | 1.4±0.1 | 0.40 |
| Time to Extubation (min) | 22.2±1.2 | 23.2±0.8 | 0.46 |
| Surgical Survival | 11/14 | 14/16 | n/a |
