# Supplementary material for: A novel ultrasound-guided mouse model of sudden cardiac arrest
Source: PLoS One. 2020 Dec 4;15(12):e0237292. doi: 10.1371/journal.pone.0237292 (PMC7717537; doi:10.1371/journal.pone.0237292)
Supplement: Attachment — Submitted filename: Response to reviewer comments.docx [file pone.0237292.s003.docx]

Response to reviewer comments:

We again thank all the reviewers for their thoughtful comments about our work. In the paragraphs below, we have responded to the comments and have indicated the specific changes made to manuscript.

***Reviewer #1****: This revised manuscript is much improved. Methodologies and results are more detailed and robust. I have no further comments.*

We are grateful for this reviewer’s contribution to this manuscript.

**Reviewer #2:** (No Response)

**Reviewer #3:** This is a very good paper, and the authors have appropriately addressed critique with 2 exceptions:

1. Although some references to "trends toward significance" have been removed, the paragraph including line 314 (in the redline version) continues to use this misleading and erroneous misuse of statistics. Overall, the claim the authors are trying to make here doesn't seem that important (it's a model of global ischemia), so the easiest thing is to just delete this paragraph. But if it's terribly important to make a poorly supported claim about global ischemia, then simply report that some animals had elevated AST/ALT/CPK/LDH, consistent with other CA models and suggesting this is a model of global ischemia, but that larger experiments would be required to fully characterize the model for this role.

Our apologies for this oversight, we have amended our language as suggested by the author. Paragraph 8 of the Discussion sections has been updated to read “…Some mice had elevated levels of CK, a non-specific marker of muscle degradation, and serum lactate, but these changes were not consistent in the SCA group. While these changes are suggestive of secondary ischemic damage as seen in post-cardiac arrest patients, larger experiments would be necessary to fully characterize these changes in our model.”

2. The third sentence in the conclusions (the one after the edited second sentence) is missing some words and is nonsensical.

Again, we apologize for this error. This sentence has been removed. The updated conclusion paragraph now reads:

“We demonstrate a novel mouse model of SCA that utilizes direct LV injection of KCl under ultrasound guidance that allows for rapid and reliable arrest with low surgical mortality. This model develops significant cardiac, kidney, and liver injury at one day as well as neurologic injury in some animals. This model lowers the barrier to entry for establishing a mouse model of SCA, which will help researchers investigate the mechanisms underlying SCA mortality.”
